# Cell Wall-Anchored Nuclease of *Streptococcus sanguinis* Contributes to Escape from Neutrophil Extracellular Trap-Mediated Bacteriocidal Activity

**DOI:** 10.1371/journal.pone.0103125

**Published:** 2014-08-01

**Authors:** Chisato Morita, Ryuichi Sumioka, Masanobu Nakata, Nobuo Okahashi, Satoshi Wada, Takashi Yamashiro, Mikako Hayashi, Shigeyuki Hamada, Tomoko Sumitomo, Shigetada Kawabata

**Affiliations:** 1 Department of Oral and Molecular Microbiology, Osaka University Graduate School of Dentistry, Osaka, Japan; 2 Department of Orthodontics and Dentofacial Orthopedics, Osaka University Graduate School of Dentistry, Osaka, Japan; 3 Department of Restorative Dentistry and Endodontology, Osaka University Graduate School of Dentistry, Osaka, Japan; 4 Department of Oral Frontier Biology, Osaka University Graduate School of Dentistry, Osaka, Japan; 5 Research Institute for Microbial Diseases, Osaka University, Osaka, Japan; Department of Biomaterials, Japan

## Abstract

*Streptococcus sanguinis*, a member of the commensal mitis group of streptococci, is a primary colonizer of the tooth surface, and has been implicated in infectious complications including bacteremia and infective endocarditis. During disease progression, *S. sanguinis* may utilize various cell surface molecules to evade the host immune system to survive in blood. In the present study, we discovered a novel cell surface nuclease with a cell-wall anchor domain, termed SWAN (streptococcal wall-anchored nuclease), and investigated its contribution to bacterial resistance against the bacteriocidal activity of neutrophil extracellular traps (NETs). Recombinant SWAN protein (rSWAN) digested multiple forms of DNA including NET DNA and human RNA, which required both Mg^2+^ and Ca^2+^ for optimum activity. Furthermore, DNase activity of *S. sanguinis* was detected around growing colonies on agar plates containing DNA. In-frame deletion of the *swan* gene mostly reduced that activity. These findings indicated that SWAN is a major nuclease displayed on the surface, which was further confirmed by immuno-detection of SWAN in the cell wall fraction. The sensitivity of *S. sanguinis* to NET killing was reduced by *swan* gene deletion. Moreover, heterologous expression of the *swan* gene rendered a *Lactococcus lactis* strain more resistant to NET killing. Our results suggest that the SWAN nuclease on the bacterial surface contributes to survival in the potential situation of *S. sanguinis* encountering NETs during the course of disease progression.

## Introduction

The oral mitis group of streptococci are part of the commensal flora in the human oral cavity and also initial colonizers in dental biofilm formation [1], [2]. Although relatively harmless members of the oral microbiota, these organisms can invade the bloodstream due to dental treatment or oral injuries, including those occurring in daily activities including eating and tooth brushing [3], [4]. Once leaving the normal habitat, the mitis group of streptococci occasionally causes systemic complications, such as bacteremia and subacute bacterial endocarditis [5], [6], [7], and have also been detected in atherosclerotic plaques [8], [9].

*Streptococcus sanguinis* is classified as a member of the mitis group of streptococci (or viridans group) [10], [11]. The species name “*sanguinis* (formerly *sanguis*)” originates from description of a set of streptococcal strains isolated from patients with subacute bacterial endocarditis [12]. Among mitis group members, epidemiological studies have shown that *S. sanguinis* is the most frequently identified species in native-valve infection cases [8], [9]. Hence, its potential virulence has been noted in the context of a causal relationship between progression of infectious cardiovascular disease and bacterial interactions with human tissues and the immune system.

Polymorphonuclear leukocytes (or neutrophils) use various strategies to combat invading microbes [13], [14], with phagocytosis being their most important function. In addition to that and exocytosis of granules, neutrophils release web-like structured neutrophil extracellular traps (NETs) to capture and kill microbes. NETs consist of DNA and antimicrobial components such as histones, LL-37, defensins, myeloperoxidase, and neutrophil elastase [15]. NET formation is induced during systemic blood infection by pathogenic bacteria, as shown in mouse infection models [16], [17]. In addition, NETs have been detected within septic thrombi, i.e., vegetation attached to heart valves of patients with infective endocarditis [18]. Thus, direct interaction between NETs and infected microbes can be speculated to occur in infected vegetation related to endocarditis. Furthermore, it has been reported that Gram-positive pathogens, including *Staphylococcus aureus* and *Streptococcus pyogenes*, escape NET killing via breakdown of NET scaffold DNA with extracellular nuclease activity [19], [20], [21].

Surface proteins covalently linked to the cell wall of Gram-positive pathogens are major determinants of virulence and participate in various biological processes, including recognition of host matrix molecules, interaction with human cells, and evasion of host immune systems. Such surface proteins of *S. sanguinis* have been implicated in the pathogenesis of infective endocarditis [22], [23], [24]. Those proteins typically possess a C-terminal cell wall sorting signal, which is comprised of a pentapeptide LPXTG motif followed by hydrophobic side chains and a positively charged tail at the C-terminus. After being guided to the bacterial surface, those proteins are processed by the transpeptidase sortase A (SrtA). SrtA catalyzes a covalent linkage between the carboxyl group of threonine in the LPXTG motif and a free amino group in the growing cell wall [25]. Thirty-three putative cell wall-anchored proteins were identified in a *S. sanguinis* strain [23], [26]. However, the exact biological role of the majority of those proteins remains elusive.

In the present study, we focused on a unique surface protein possessing that cell wall sorting signal and a putative nuclease domain. Utilizing a recombinant procedure, we confirmed and characterized its nuclease activity. Furthermore, we generated a deletion mutant and examined evasion of NET killing by *S. sanguinis*.

## Materials and Methods

### Bacterial strains and culture conditions

*S. sanguinis* strain SK36 (kindly provided by Dr. Kilian) [27] and its derivatives were routinely cultured in Todd-Hewitt broth (TH, Becton Dickinson, NJ, USA) at 37°C. For a deoxyribonuclease (DNase) assay using agar plates, *S. sanguinis* strains as well as *S. oralis* NCTC 11427T/SK23 [27], *S. mutans* MT8148 [28], *S. salivarius* HHT [29], *S. parasanguinis* ATCC 903 [27], and *S. sobrinus* MT10186 [30] were cultured in Brain Heart Infusion (BHI) broth (Becton Dickinson). The *Escherichia coli* strain TOP10 (Life Technologies, CA, USA) served as a host for derivatives of pSET6s and pAT18 [31], [32]. The *E. coli* strain XL10-gold (Stratagene, CA, USA) was utilized as a host for the pQE30 derivatives (Qiagen, Germany). *E. coli* strains were cultured in Luria-Bertani (LB, Sigma Aldrich, MO, USA) medium at 37°C with constant agitation. *Lactococcus lactis* NZ9000 (kindly provided by Dr. Poolman) and its derivatives were grown in M17 broth (Becton Dickinson) containing 0.5% glucose (M17G, Wako, Japan) at 28°C. To select mutant strains, antibiotics were added to the media at the following concentrations: ampicillin (Wako); 100 µg/ml for *E. coli*, chloramphenicol (Sigma Aldrich); 10 µg/ml for *E. coli* and 5 µg/ml for *S. sanguinis*, and erythromycin (Sigma Aldrich); 150 µg/ml for *E. coli* and 1 µg/ml for *L. lactis*.

### Evaluation of extracellular DNase activity of oral streptococcal species

Extracellular DNase activity of the streptococcal strains was examined using a method previously described by Jefferies *et al*., with some modification [33]. Briefly, a 5-µl aliquot of an overnight culture was spotted onto BHI agar plates containing 2 mg/ml of salmon sperm DNA (Wako), 0.5 mM MgCl_2_, and 0.5 mM CaCl_2_. The plates were incubated at 37°C for 72 h, and flooded with 1 M of HCl. DNA-digested areas were indicated by a clear halo around colony clusters.

### Construction and purification of a series of recombinant SWAN

Recombinant N-terminally His_6_-tagged SWAN protein (rSWAN) was prepared using the *E. coli*-expression vector pQE30. Briefly, *swan* DNA encompassing the entire protein without the putative signal sequence and C-terminal sorting signal was amplified by PCR using the primers rSWANF 5′-gcggatccgaagaagcggtaagttcgtcg-3′ and rSWANR 5′-gccccgggggtcttcgggagtcccttctt-3′. The amplicon was digested with *Bam*H I and *Sma* I, and cloned into pQE30 (Qiagen). All primers were designed using the genome sequence of *S. sanguinis* strain SK36 (Genbank accession number: CP000387.1) [26]. Hyperexpression of rSWAN was induced with 1 mM of isopropyl-β-D-thiogalactopyranoside (Wako) at 37°C for 5 h. The cells were lysed with 100 µg/ml of lysozyme (Wako) and intermittent sonication. Then, recombinant protein was purified from the lysates using a QIAexpress protein purification system (Qiagen) according to the manufacturer's instructions. Eluted protein was ultrafiltrated with Amicon Ultra 3K filter units (Millipore, MA, USA). The concentration was determined using a BCA protein assay kit (Pierce, IL, USA). Purity and integrity of the recombinant proteins were examined by SDS-PAGE and following Coomassie Brilliant Blue staining.

### Biochemical analysis of rSWAN

To examine the DNase activity of rSWAN, 0.3 µg of λDNA (Promega, WI, USA) was incubated with 0.1 µg of rSWAN in reaction buffer containing 50 mM tris (hydroxymethyl) aminomethane (Tris)-HCl (pH 7.5), 15 mM NaCl with or without 1 mM Ca*^2+^*, and 1 mM Mg*^2+^* at 37°C for 1 h. Following electrophoresis in 1% Tris-borate-ethylenediaminetetraacetic acid (EDTA, TBE) agarose gel, DNA was stained with 1 µg/ml ethidium bromide (EtBr, Wako) and visualized under UV light.

For examination of the optimal concentrations of Ca*^2+^* and Mg*^2+^*, 0.3 µg λDNA was incubated with 0.1 µg rSWAN in the same reaction buffer with serially diluted Ca*^2+^* or Mg*^2+^* at 37°C for 30 min. After electrophoresis, gel images were analyzed with Image J software (http://rsbweb.nih.gov/ij/) and % DNA digestion was calculated.

To test the ribonuclease (RNase) activity of rSWAN, 10 µg of total RNA purified from the human keratinocyte cell line HaCaT [34] with Trizol and a PureLink RNA mini kit (Life technologies) was incubated with 0.1 µg of either rSWAN or recombinant T6 streptococcal pilus protein (rT6 pilus protein) [35] in reaction buffer containing 1 mM Ca*^2+^* and 1 mM Mg*^2+^* at 37°C for 1 h. To exclude the possibility of RNase contamination, rT6 pilus protein purified in the same manner as rSWAN was also tested as a negative control.

For testing the SWAN cleavage preference of DNA forms, multiple forms of phage-derived DNA (NEB, MA, USA) [36], including φX174 RF I DNA (double-stranded circular), φX174 RF II DNA (double-stranded nicked circular), and φX174 Virion DNA (single-stranded circular), were incubated with 0.1 µg of rSWAN. The reaction was stopped with EDTA at a final concentration of 10 mM, which was followed by electrophoresis and EtBr staining.

### Neutrophil isolation and NET cleavage assay

Neutrophils were isolated from freshly drawn blood of healthy donors. Red blood cells were sedimented from heparinized whole blood by mixing with a saline solution containing 3% Dextran T-500 (GE Healthcare, UK). Then, the upper layer containing leukocyte-rich plasma was subjected to density centrifugation in Ficoll-Paque solution (GE Healthcare). The resultant pellet was washed with hypotonic lysis buffer, containing 0.15 M NH_4_Cl, 10 mM KHCO_3_, and 0.1 mM EDTA, to lyse residual red blood cells, followed by washing with cold phosphate-buffered saline (PBS). Cells were suspended in RPMI medium (Wako) containing 2% human serum albumin (HSA, Sigma Aldrich) and the number of neutrophils was enumerated using a hemocytometer. Next, 2×10^5^ cells were seeded into each well of poly-_L_-lysine (Sigma Aldrich)-coated 8-well chamber slides (Nalge Nunc, NY, USA) and incubated for 30 min to allow the cells to attach to the well bottom. For NET induction, cells were stimulated with 200 nM of phorbol 12-Myristate 13-Acetate (PMA, Wako) and incubated for 2 h. The medium was replaced with buffer containing 0.1 M 4-(2-hydroxyethyl)-1-piperazineethanesulfonic acid, pH 7.5, 150 mM NaCl, 2% HSA, 1 mM MgCl_2_, and 1 mM CaCl_2_, then cells were incubated with or without rSWAN at a final concentration of 40 µg/ml. After a 1-h incubation, cells were fixed with 4% paraformaldehyde in PBS for 20 min and permealized with PBS containing 0.05% Triton X-100 (Wako) for 20 min at room temperature. The cells were then blocked overnight with 1% bovine serum albumin (Sigma Aldrich) in PBS at 4°C and reacted with a goat anti-human elastase polyclonal antibody (1∶2000, Santa Cruz Biotechnology, CA, USA) for 1 h at room temperature. After washing with PBS, the cells were incubated with Alexa Fluor 594-conjugated anti-goat IgG (1∶1000, Molecular Probes, OR, USA) for 1 h at room temperature. Finally, the slides were enclosed with ProLong Gold Antifade Reagent with DAPI (Life technologies) and observed using a Carl Zeiss Axioplan 2 fluorescent microscope system.

### Transformation of *S. sanguinis* SK36

*S. sanguinis* SK36 cells were cultured overnight in TH broth, then diluted 1∶100 in the same medium containing 10% heat-inactivated horse serum (SAFC Biosciences, MO, USA). The cultures were incubated at 37°C until the *A*_600_ value reached 0.1. After adding a synthetic competence stimulating peptide (DLRGVPNPWGWIFGR, purity 98.8%, SIGMA) [37] at a concentration of 1 µg/ml, the cultures were incubated at 37°C for 10 min. Finally, plasmids were added at concentrations of 1 µg/ml and further incubated at 37°C for 8 h, followed by plating on TH agar plates containing chloramphenicol.

### Construction of mutant strains of *S. sanguinis*

An in-frame deletion mutant of the *swan* gene (Delswan) and its revertant (Wr) were generated from wild-type (WT) *S. sanguinis* SK36, using a temperature sensitive shuttle vector, pSET6s, as previously described [35]. The constructed plasmid was transformed into SK36 as described above and grown in the presence of chloramphenicol. After integration of the plasmid into the chromosome via first allelic replacement, cells were cultured at 28°C without chloramphenicol to induce the second allelic replacement. Deletion of the *swan* gene was confirmed by site-specific PCR using purified genomic DNA. To rule out the effects of secondary mutations, a clone possessing the WT allele that arose during the course of mutagenesis was also utilized as a revertant strain (Wr).

### Preparation of antiserum against rSWAN

Mouse antiserum against SWAN was raised by immunizing BALB/c mice (Charles River Japan, 5 weeks old, female) with a purified recombinant N-terminal fragment of SWAN (33-357 amino acids), as previously described [38]. Briefly, mice were vaccinated intradermally with a mixture of 200 µg of recombinant proteins and TiterMax gold adjuvant (CytRx, CA, USA), followed by 3 boosts with 200 µg of recombinant protein with the adjuvant at 1-week intervals. Whole blood samples were collected via the orbital venous plexus and serum was utilized for immunoblot analyses, as described below.

### Detection of SWAN in cell wall fraction of *S. sanguinis* cells

*S. sanguinis* cells were grown overnight in TH medium and washed twice with PBS, then resuspended in protoplasting buffer containing 0.1 M KPO_4_, pH 6.2, 0.3 M raffinose, 10 mM MgCl_2_, complete EDTA-free protease inhibitors (Roche, Switzerland) and 200 units/ml of mutanolysin (Sigma Aldrich). The cells were incubated at 37°C for 3 h with mild rotation. Protoplasts were sedimented by centrifugation at 20,000×*g* for 20 min. Supernatants were collected as cell wall fractions, and proteins in the fractions were separated by SDS-PAGE and blotted onto polyvinylidene difluoride membranes (Millipore). Each membrane was blocked with a casein-based solution (Megmilk Snow Brand, Japan) and incubated with mouse anti-SWAN antiserum (1∶2000) for 1 h at room temperature, followed by washing steps and incubation with an anti-mouse IgG horseradish peroxidase-conjugated secondary antibody (Cell Signaling Technology, MA, USA). The membranes were then washed and developed with a Western blotting substrate (Pierce).

### NET infection assay

We performed a NET infection assay as previously reported, with minor modifications (15). Human neutrophils were suspended in RPMI containing 10% fetal bovine serum (FBS, SAFC Biosciences) and seeded into wells of a 96-well culture plate (IWAKI, Japan) at a concentration of 5×10^4^ cells/well. After a 30-min incubation, NETs were induced with 200 nM of PMA for 2 h. Cells were then treated with cytochalasin D (Sigma Aldrich) at a concentration of 20 µg/ml to prevent phagocytosis. Following a 30-min incubation, cells were infected with *S. sanguinis* at a multiplicity of infection (MOI) of 5. Then, cells were sequentially lysed by adding 100 µl of lysis buffer containing 460 mM NaCl and 0.05% Triton X-100, and serial dilutions were plated on TH agar plates. Bacteria were grown at 37°C for 24 h and survival rate was calculated by counting the number of colonies.

### Heterologous expression of *swan* in *L. lactis*

For heterologous expression of *swan* in *L. lactis*, the *swan* structural gene and putative transcriptional terminator were amplified by PCR, and cloned into the shuttle vector pAT-P*gyrA* [38]. The resultant plasmid was transformed into *L. lactis* NZ9000 [39] and the transformant (Lswan) was grown in the presence of erythromycin. The strain was also transformed with an empty plasmid (Mock) and utilized as a negative control in functional assays.

The surface display of SWAN in Lswan was examined using Western blot analysis. Transformants were grown in 10 ml of M17G to the exponential phase (*A*_600_ = 0.6) and washed twice with PBS. Cells were then suspended in 100 µl of protoplasting buffer containing 0.1 M KPO_4_, pH 6.2, 40% sucrose, 10 mM MgCl_2_, complete EDTA-free protease inhibitors (Roche), and 200 units/ml of mutanolysin (Sigma Aldrich), and incubated at 37°C for 1 h in a rotator. Protoplasts were sedimented by centrifugation at 20,000×*g* for 10 min and the supernatants were used as cell wall fractions for Western blot analysis.

The DNase activity of *L. lactis* was examined using the aforementioned DNA-containing agar plates after incubation for 24 h. A NET infection assay was also performed using the recombinant *L. lactis* strains as described above, except for being performed at an MOI of 10.

### Statistical analysis

The significance of differences between the means of groups was evaluated using Scheffe's test with one-way ANOVA and Student's *t*-test, with StatView software (SAS Institute, NC, USA). A confidence interval with a *p* value of <0.05 was deemed to indicate significance.

### Ethics statement

Mouse immunizing experiments for antiserum preparation followed a protocol approved by the Animal Care and Use Committee of Osaka University Graduate School of Dentistry. Human venous blood was obtained from healthy volunteers after obtaining written informed consent according to a protocol approved by the institutional review board of Osaka University Graduate School of Dentistry.

## Results and Discussion

### Extracellular nuclease activity of *S. sanguinis*

**Figure 1:**
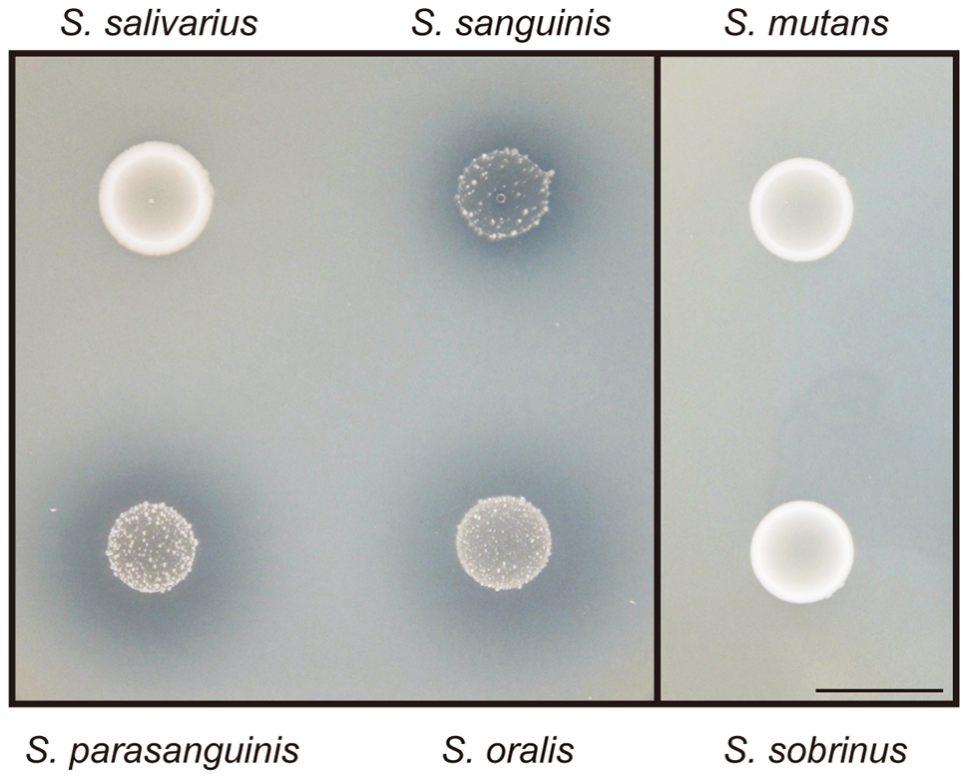
DNA digestion around growing colonies of streptococcal species. The DNase activities of oral streptococcus strains were examined using BHI agar plates containing salmon sperm DNA. The plates were incubated for 72-digested DNA. Halos seen around colonies reflect DNA digestion. The following strains were tested: *S. sanguinis*, SK36; *S. oralis*, NCTC 11427T/SK23; *S. mutans*, MT8148; *S. salivarius*, HHT; *S. parasanguinis*, ATCC 903; *S. sobrinus*, MT10186. Bar, 1 cm.

Since many Gram-positive bacterial pathogens utilize secreted DNase to exert their virulence [19], [20], [21] and DNase secretion from oral streptococci has not been investigated in detail, we examined whether viable organisms of oral streptococci show extracellular DNase activity. *S. sanguinis* SK36 and the tested oral streptococcus strains, including *S. salivarius* HTT, *S. mutans* MT8148, *S. parasanguinis* ATCC 903, *S. oralis* SK23, and *S. sobrinus* MT10186, were cultured in indicator BHI agar plates containing salmon sperm DNA. Unexpectedly, a clear zone around growing colonies was notably observed for *S. sanguinis* and other closely related species, i.e., *S. parasanguinis* and *S. oralis*, while no such zone was seen in the *S. salivarius*, *S. mutans*, and *S. sobrinus* cultures (Fig. 1). These observations indicated that *S. sanguinis* SK36 and other tested mitis group streptococcal strains secrete DNase into extra-cytoplasmic space. Since these DNase-positive species have been notably implicated in cardiovascular diseases [5], extracellular DNase activities of *S. sanguinis* and *S. oralis* may be involved in disease progression.

### Identification of putative DNase of *Streptococcus sanguinis*

**Figure 2:**
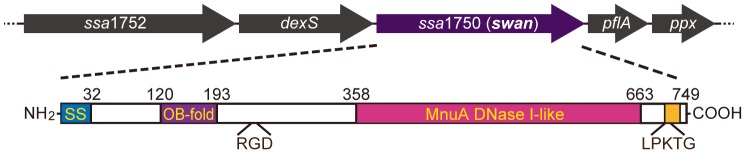
Chromosomal *swan* gene locus and SWAN domain organization. A genetic map around *ssa1750* (*swan*) from *Streptococcus sanguinis* strain SK36 is shown. Gene designations or gene tag numbers (*ssa*) are indicated inside the genes (arrows). The domain organization of SWAN is also shown. The positions of the putative signal sequence (SS), the cell wall sorting signal containing a Sortase A-recognizable LPKTG sequence, an RGD sequence, oligosaccharide/nucleotide binding fold (OB-fold), and an MnuA DNase 1-like domain are depicted with start and end positions of the amino acid residue numbers from the N-terminus.

As a first step to identify the responsible DNase of *S. sanguinis* strain SK36, we searched putative surface-expressed proteins in a genome database [26]. Of genes encoding cell wall-anchored proteins, we found a noteworthy plausible candidate, the *ssa1750* gene encoding a 749 amino acid protein. The *ssa1750* gene is located between a *pflA* encoding putative pyruvate formate-lyase-activating enzyme and *dexS* encoding dextran glucosidase in the chromosomal DNA of SK36 (Fig. 2). In the upstream region of the structural gene, a putative ribosomal binding site and promoter sequence were detected, while the deduced transcriptional terminator was also observed to be located just downstream of the gene. Therefore, it is likely that *ssa1750* is transcribed in a monocistronic manner. The SSA1750 protein contains the putative signal peptidase-cleaving site between Ala32 and Glu33, as shown using the SignalP 4.1 server (http://www.cbs.dtu.dk/services/SignalP/), and a cell wall sorting signal with the SrtA-recognizable sequence LPKTG (Fig. 2). A domain search using the conserved domain database (CDD) [40] revealed an MnuA DNase1-like domain (CD10283) [41], which belongs to a subfamily of the DNase I family comprising the EEP (endonuclease/exonuclease/phosphatase) domain superfamily. Moreover, an N-terminal oligosaccharide/nucleotide binding fold (OB-fold) similar to the OB folds of *Bacillus subtilis* nuclease YhcR (CD04486) (Fig. 2) [42] was recognized. These features prompted us to frame a hypothesis that SSA1750 functions as a DNase. Between these domains, an RGD sequence was noted, which is the minimum unit of fibronectin to bind integrin [43]. Although we did not address the biological significance of the RGD sequence in this study, interaction between human integrin and SSA1750 is expected.

Since a clear halo was observed around the colonies of *S. parasanguinis* and *S. oralis* (Fig. 1), we also searched for a gene similar to *ssa1750* in their genome sequences (GenBank IDs FR720602.1 and CP002843.1). Using a BLAST search against the genome, a gene homologous to *ssa1750* was detected in the genome of *S. parasanguinis* ATCC 15912 (locus tag HMPREF0833_10979). However, the homologue was not present in *S. oralis* Uo5, indicating the presence of a different kind of secreted DNase. *S. oralis* is closely related to the human pathogen *S. pneumoniae*, which secretes surface-exposed DNase, a DNA-entry endonuclease EndA [44], and the EndA homologue was found to be encoded in the genome of *S. oralis* (locus tag SOR_0267). Therefore, the presence of a halo around the *S. oralis* colony was attributed to probable DNase activity of the EndA-like protein, though EndA is not a homologue of SSA1750. A BLAST search using the SSA1750 amino acid sequence against a non-redundant database indicated that a variety of streptococcal species possess a protein homologous to SSA1750, including *S. gordonii*, *S. anginosus*, *S. constellatus*, *S. intermedius*, *S. suis* [45], *S. merionis*, *S. henryi*, *S. iniae*, *S. equi*, *S. dysgalactiae*, *S. canis*, and *S. pyogenes* [46].

### Characterization of rSWAN enzymatic activity

**Figure 3:**
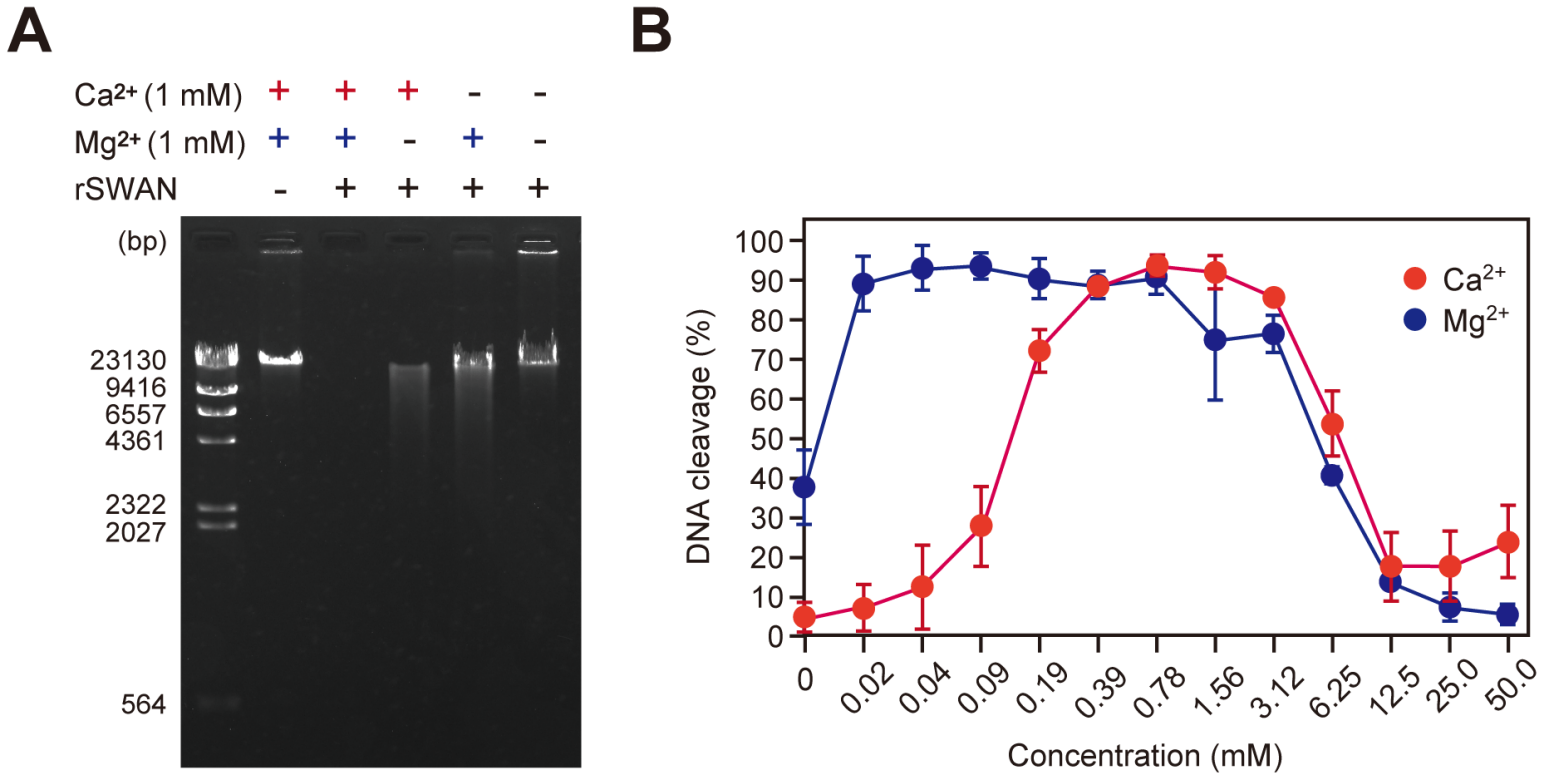
Efficient DNase activity of rSWAN requires Ca^2+^ and Mg^2+^. (A) λDNA (0.3 µg) was incubated with recombinant SWAN (rSWAN, 0.1 µg) with or without CaCl_2_ (1 mM) and MgCl_2_ (1 mM) at 37°C for 1 h. Following electrophoresis, DNA was stained with ethidium bromide and visualized under UV light. The sizes of the λDNA *Hin*d III digest markers are indicated on the left. (B) λDNA (0.3 µg) was incubated with rSWAN (0.1 µg) with varying concentrations of either Ca^2+^ (red circles) or Mg^2+^ (blue circles) at 37°C for 30 min. Using gel images and Image J software, densitometric analyses were performed to calculate % DNA cleavage. Values shown represent the average ± SE of 3 independent experiments.

To ascertain the DNase activity of SSA1750 protein, the purified recombinant protein was incubated with double-stranded linear *λ*DNA [47] in the presence of Ca*^2+^* and Mg*^2+^*. DNA was completely digested by rSSA1750 at 37°C for 1 h, as revealed by gel electrophoresis (Fig. 3A). Based on those as well as the following findings in our study, SSA1750 was designated as SWAN.

In the absence of both Ca*^2+^* and Mg*^2+^*, the DNase activity of recombinant SWAN (rSWAN) against *λ*DNA was nearly completely undetectable (Fig. 3A). Depletion of either Ca*^2+^* or Mg*^2+^* from the reaction also partially decreased the activity. Thus, efficient DNase activity requires both Ca*^2+^* and Mg*^2+^*. Since requirement of a divalent metal ion is a feature of DNase I family proteins, a similar catalytic property was postulated to exist in SWAN. When a 2-fold dilution series of Ca*^2+^* and Mg*^2+^* was tested for the reaction after incubation for 30 min, densitometric analyses showed that the concentrations of Ca*^2+^* and Mg*^2+^* that allowed more than 70% DNA cleavage were approximately 0.19–3.12 and 0.02–3.12 mM, respectively (Fig. 3B), which are within the range of concentrations in human saliva and serum [48], [49], [50], [51]. We also examined optimal pH for the activity. The optimal pH and acid stability of DNase I family members vary, which has been postulated to arise from adaptation to the surrounding environmental condition and indicate the evolutionary history [52]. rSWAN showed DNase activity with a broad pH range (pH 5.5–9.0) and exhibited its maximum activity at approximately neutral pH (data not shown). Nevertheless, it is suggested that SWAN is able to cleave DNA in environments encountered by *S. sanguinis*, such as the oral cavity and bloodstream, where pH is maintained at an approximately neutral level.

**Figure 4:**
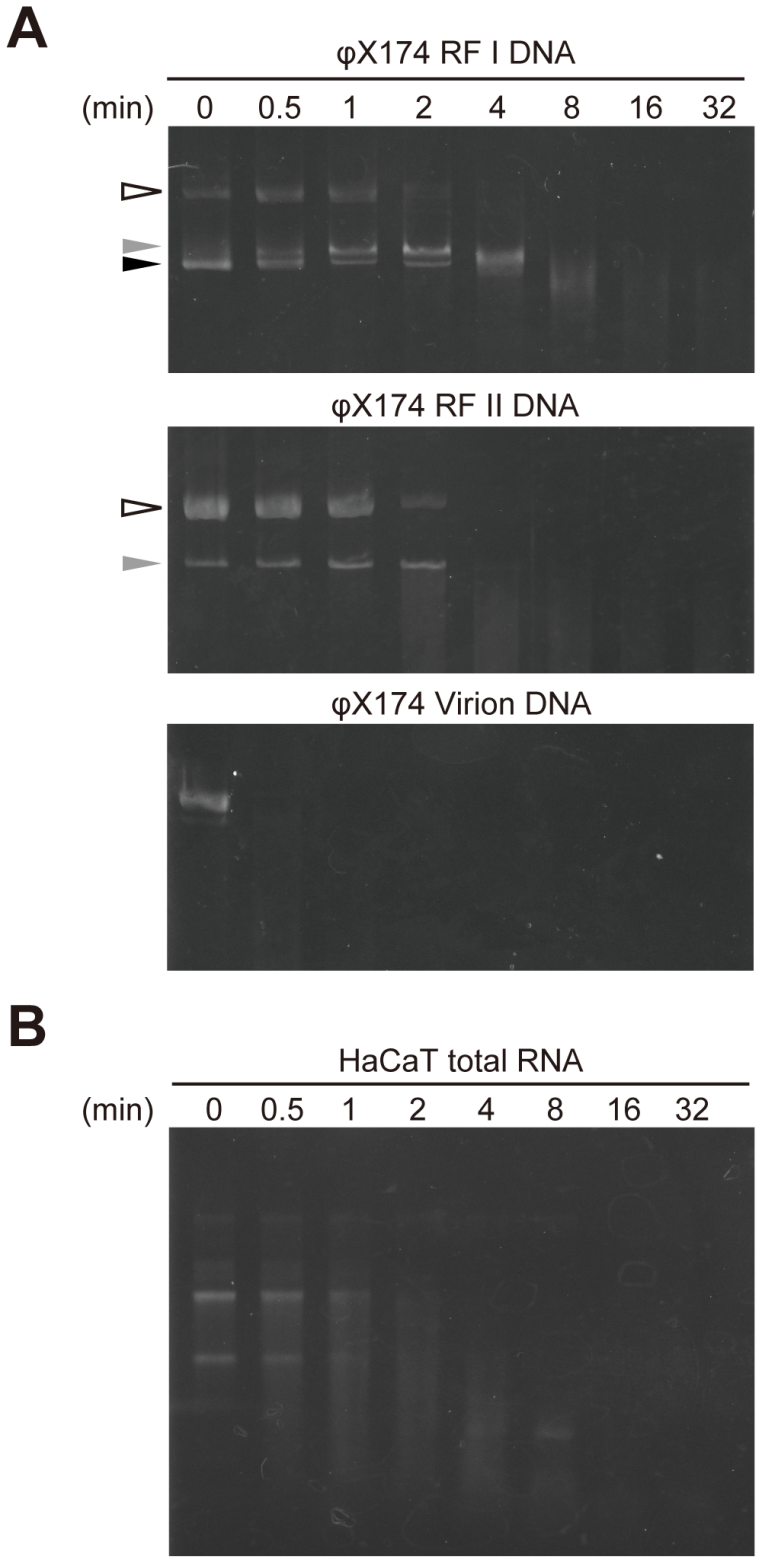
Nuclease activity of rSWAN against variable forms of viral DNA and RNA. (A) The substrate specificity and preference of rSWAN were examined using φX174RF I DNA (double-stranded, mainly super coiled circular), φX174 RF II DNA (double-stranded, mainly nicked circular), and φX174 DNA (single-stranded, mainly circular). Open arrowhead, open circular form; black arrowhead, super coiled form; gray arrowhead, linear form. (B) Total RNA from human keratinocytes was incubated with rSWAN at 37°C for the indicated time periods.

Next, the specificity and preference of the DNA substrate for rSWAN were examined using multiple forms of DNA derived from bacteriophage φX174 *am*3 *cs*70 [36], including double-stranded super coiled circular, double-stranded nicked circular, and single-stranded circular DNA (Fig. 4A). DNase I preferentially hydrolyzes double-stranded DNA and B-form DNA is the best substrate with some sequence preference [53]. As for SWAN, all DNA substrates were readily digested with rSWAN. Double-stranded super coiled and nicked circular DNAs were completely digested after 4–8 min, whereas single-stranded circular DNA was already digested after 30 sec, indicating a propensity of SWAN to efficiently digest single-stranded DNA.

Using total RNA from a human keratinocyte cell line, we also examined whether rSWAN digests RNA (Fig. 4B). The RNA was efficiently digested by rSWAN and no band was detected after 16 min of incubation. To exclude the possibility of RNase contamination during rSWAN preparation, a recombinant T6 streptococcal pilus protein [35] was also tested as a negative control. In contrast to the rSWAN-treated samples, distinguishable digestion was not detected (data not shown). Thus, in addition to DNase activity, SWAN also showed RNase activity, confirming that SWAN is a nuclease that recognizes a broad range of nucleic acid substrates.

**Figure 5:**
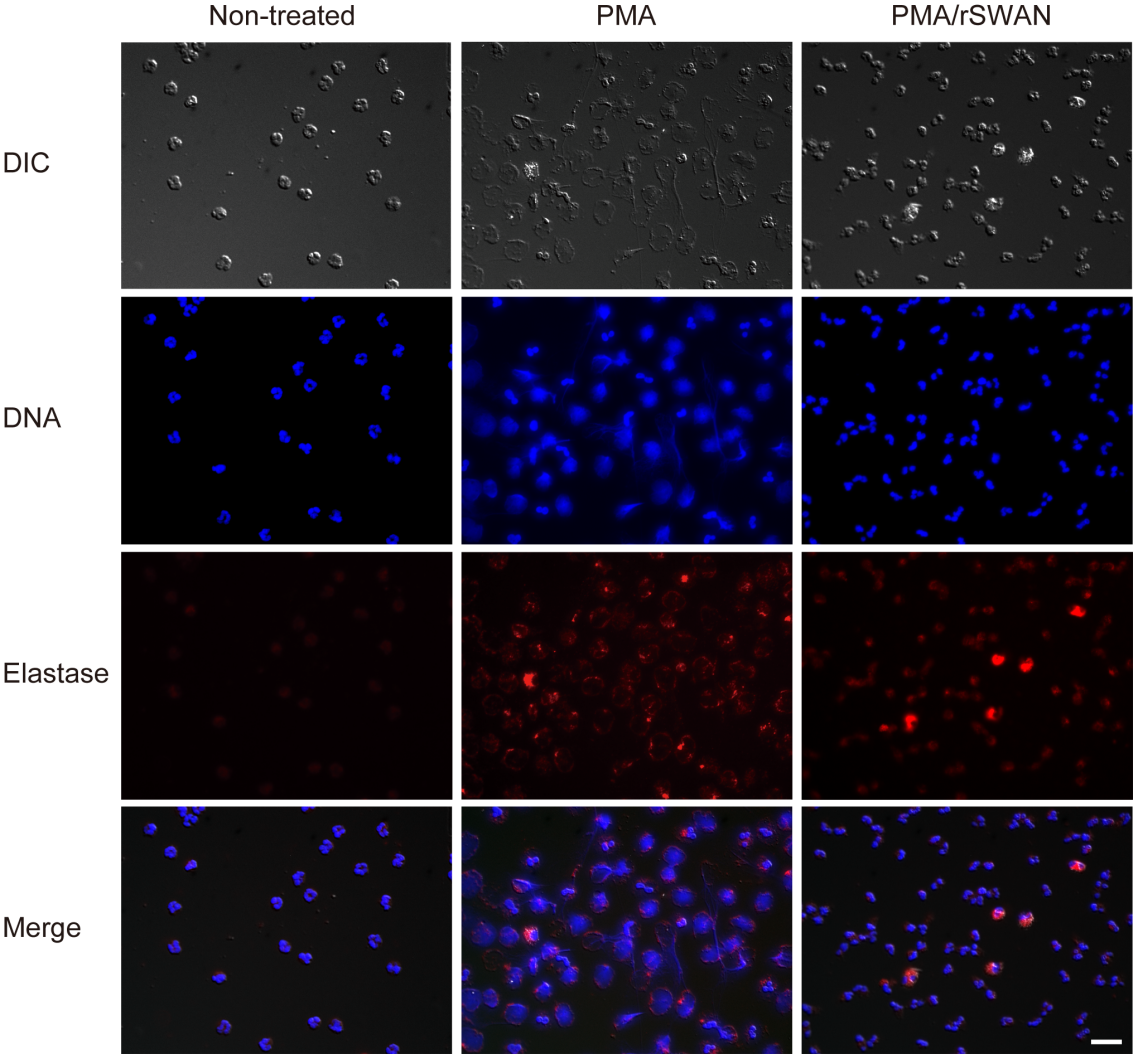
rSWAN cleaves NET DNA. Neutrophils were isolated from heparinized human blood and NETs were induced with PMA. NETs were incubated with or without rSWAN (40 µg/ml) at 37°C for 1 h. After fixation and permealization, neutrophil elastase was labeled with rabbit anti-human elastase IgG and an Alexa Fluor 594-conjugated secondary antibody, and DNA was stained with DAPI. Neutrophils without treatment with PMA and rSWAN were utilized as a control. The slides were observed using fluorescent microscopy. Bar, 20 µm.

Secreted streptococcal DNase has been shown to digest the DNA scaffold of NETs [54], [55], [56], thus we performed *in vitro* NET induction with PMA and examined whether rSWAN digests NET DNA (Fig. 5). Neutrophils from healthy donors, of which elastase and DNA were fluorescently labeled, showed a lobulated nucleus and faint staining of elastase (Fig. 5, left column). After treatment with PMA, the nuclei of neutrophils became decondensed and lost lobulation (Fig. 5, middle column). Extracellular DNA, i.e., NETs, released from the cells was also observed, as was extracellular staining of elastase. In contrast, extracellular DNA staining was not seen in activated neutrophils treated with rSWAN (Fig. 5, right column). Therefore, it is likely that rSWAN digests extracellular DNA released from activated neutrophils.

### SWAN localized on cell surface of *S. sanguinis* SK36

**Figure 6:**
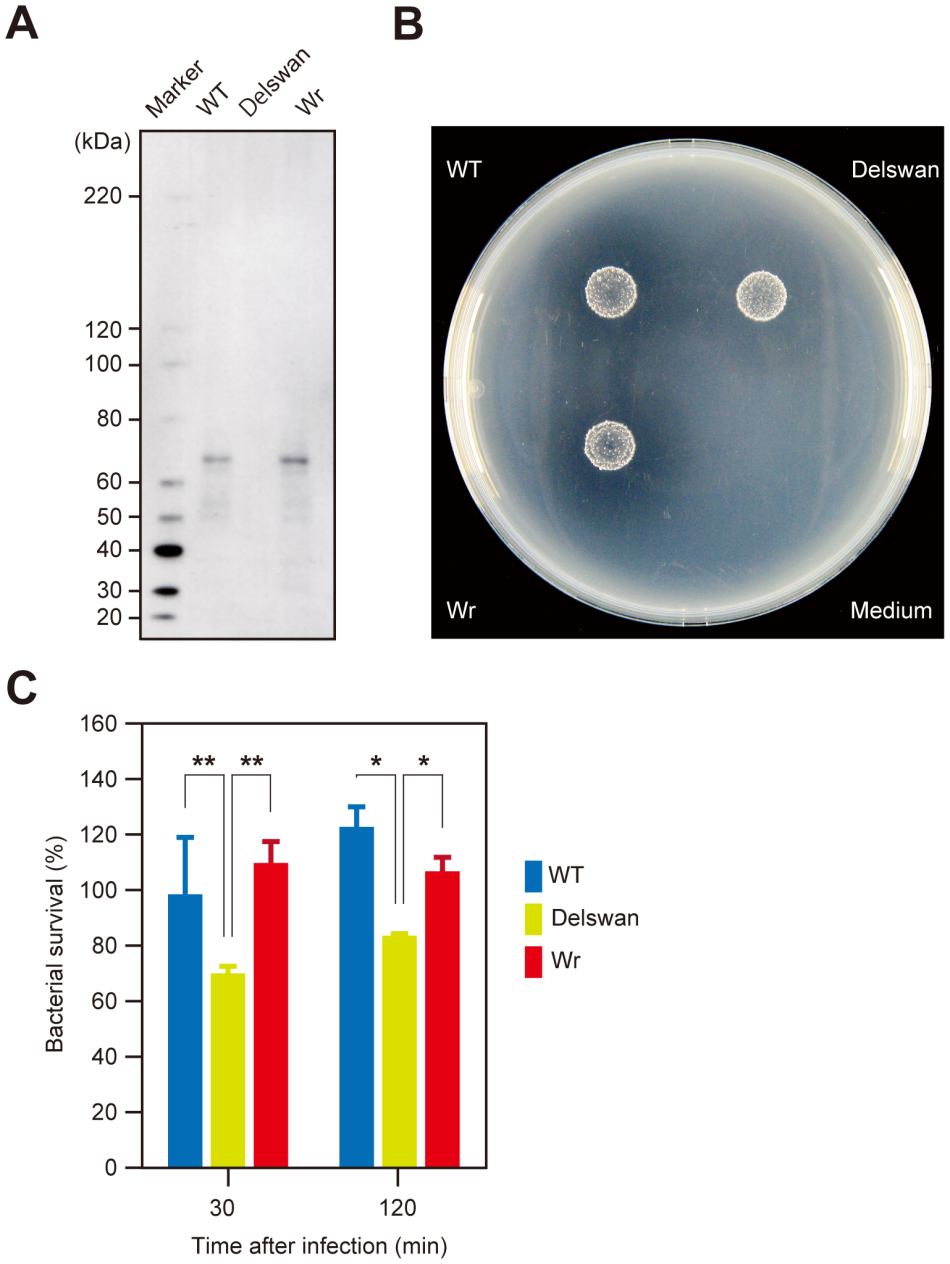
SWAN contributes to escape from bacteriocidal effects of NETs. (A) Total proteins in the cell wall fractions of *S. sanguinis* SK36 (WT), the *swan* deletion mutant (Delswan), and the revertant strain (Wr) were immunoblotted with mouse antiserum against the N-terminal recombinant fragment of SWAN. (B) The DNase activities of WT, Delswan, and Wr on BHI agar plates containing DNA were examined as described in Fig. 1. (C) *S. sanguinis* strains grown to the late-exponential phase were exposed to NETs at an MOI of 5. After the indicated times, neutrophils were lysed and the lysates were plated on Todd-Hewitt agar plates. The survival rate of *S. sanguinis* strains is shown as % inoculated colony forming units. Data were pooled from 4 independent experiments performed in triplicate and the values are shown as the mean ± SD. Statistical analysis was performed using one-way ANOVA and Scheffe's test. A confidence interval of *p*<0.05 was considered to be significant. *p<0.01, **p<0.05.

To analyze the *in vivo* function and localization of SWAN, an in-frame deletion mutant of *swan* (Delswan) was constructed on a background of strain SK36 (WT). As a control, the revertant mutant that arose during the mutant construction and possessed the WT allele (Wr) was also tested. The growth rates of Delswan and Wr were comparable to that of WT (data not shown). Utilizing these strains, the surface display of SWAN was examined using immunoblot analysis with the cell wall fractions and mouse antiserum against the truncated N-terminal fragment of rSWAN (Fig. 6A). In the WT and Wr fractions, a band was detected with a size of approximately 70 kDa, which is nearly consistent with the size of mature SWAN at 73.96 kDa. In contrast, the band disappeared in the Delswan fraction, confirming *swan* deletion. These findings suggested that SWAN is surface associated and probably cross-linked to the cell wall via the action of SrtA. DNase activity of the strains was then examined, as shown in Figure 1. As compared with the WT and revertant strains, the clear zone reflecting DNA digestion was smaller and more ambiguous around the Delswan colonies, indicating that SWAN functions as a major nuclease on the cell surface (Fig. 6B).

### SWAN contributes to evasion of antimicrobial activity of NETs

**Figure 7:**
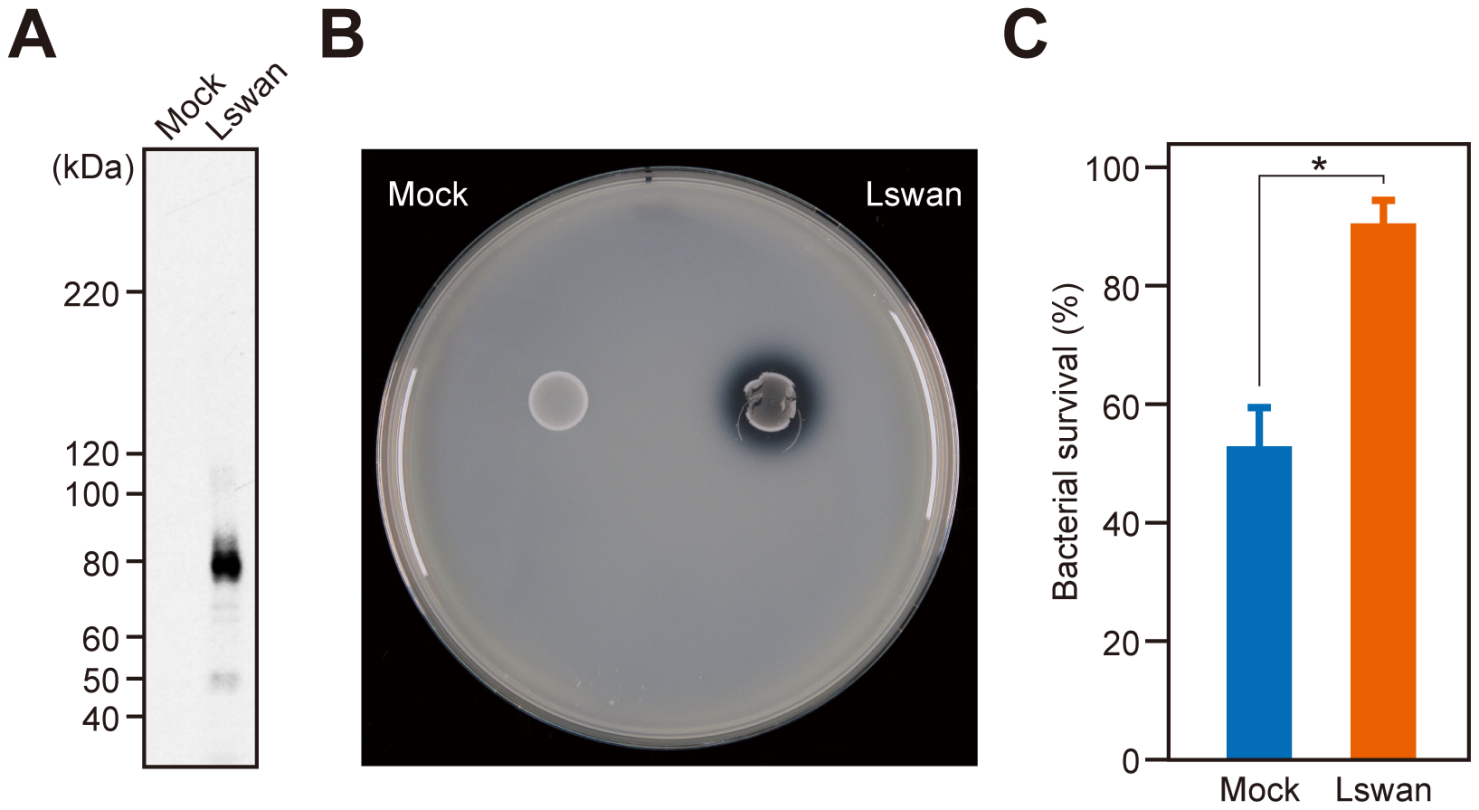
Heterologous expression of *swan* in *Lactococcus lactis*. (A) Immunoblot analysis for detection of SWAN in cell wall fractions of empty vector-transformed *L. lactis* (Mock) and *swan*-expressing (Lswan) strains. (B) The DNase activities of Mock and Lswan were examined by culturing in DNA-containing BHI agar plates for 24 h. (C) Human NETs were exposed to Mock or Lswan at an MOI of 10 for 2 h. The survival rate of recovered bacteria is shown as % inoculated colony forming units. Data were pooled from 3 independent experiments performed in triplicate and the values are shown as the mean ± SD. Statistical analysis was conducted with Student's *t*-test. *p<0.01.

We also investigated whether the nuclease activity of SWAN renders *S. sanguinis* more resistant to the antimicrobial activity of NETs (Fig. 6C). Human neutrophils were stimulated with PMA to induce NETs, followed by infection with WT, Delswan, or Wr at an MOI of 5. After 30 min of infection, the recovered bacterial count of Delswan was modestly yet significantly decreased, as compared with WT and Wr. The same was also observed after 2 h of infection. Thus, the DNase activity of SWAN likely attenuates the bacteriocidal activity of NETs. This model was corroborated by findings of an additional experiment using a heterologous *L. lactis* expression system. In the bacterial surface fraction of the recombinant *L. lactis* NZ9000 designed to express *swan* (Lswan) under the *gyrA* promoter, SWAN was readily detected by immunoblot analysis. In contrast, no band was visible in the fraction of a strain transformed with an empty shuttle vector (Mock, Fig. 7A). The DNase activity of Lswan, but not of Mock, was clearly indicated by halos around the colonies (Fig. 7B). When these strains were infected with NETs at an MOI of 10 for 2 h, the count of recovered Mock was reduced to approximately 50% of the inoculated strain (Fig. 7C). In contrast, 90% of inoculated Lswan survived under the same condition. Together with results from examinations of *S. sanguinis*, our findings strongly suggest that SWAN nuclease activity augments bacterial survival against NETs.

In summary, the present study demonstrated that the oral commensal *S. sanguinis* produces a novel wall-anchored broad-range nuclease termed SWAN. Biochemical assays revealed that divalent cations, such as Mg^2+^ and Ca^2+^, are required for optimum enzymatic activity. As a biological consequence of SWAN, our results indicate that nuclease activity contributes to evasion of *S. sanguinis* from NET killing. Moreover, since this streptococcal surface protein possesses the RGD sequence, the minimum unit of fibronectin to bind integrin [36], it is possible that SWAN exerts a function in addition to nuclease activity. The exact role of SWAN in the oral cavity and progression of cardiovascular disease will be examined in a future study.
